# Cationic biopolymer decorated Asiatic Acid and *Centella asiatica *extract incorporated liposomes for treating early-stage Alzheimer’s disease: An *In-vitro* and *In-vivo* investigation

**DOI:** 10.12688/f1000research.128874.1

**Published:** 2022-12-19

**Authors:** Akhilesh Dubey, Namdev Dhas, Anup Naha, Usha Rani, Ravi GS, Amitha Shetty, Chaithra R Shetty, Srinivas Hebbar

**Affiliations:** 1 Nitte (Deemed to be University), NGSM Institute of Pharmaceutical Sciences, Department of Pharmaceutics, Mangalore, Karnataka, India; 2 Department of Pharmaceutics, Manipal College of Pharmaceutical Sciences, Manipal Academy of Higher Education, Manipal, Karnataka, 576104, India; 3 Department of Health Innovation, Prasanna School of Public Health, Manipal Academy of Higher Education, Manipal, Karnataka, 576104, India; 4 Nitte (Deemed to be University), NGSM Institute of Pharmaceutical Sciences, Department of Pharma Chemistry, Mangalore, Karnataka, India

**Keywords:** Asiatic acid, Centella asiatica extract, Neuroprotective, Chitosan-coated liposome, Alzheimer's disease, Oral bioavailability

## Abstract

**Background:** Asiatic acid (AA) is a naturally occurring triterpenoid derivative of *Centella asiatica* (CA) with neuroprotective effect. The study aimed to design an ideal oral drug delivery system to treat Alzheimer's disease (AD) and develop chitosan-embedded liposomes comprising an extract of CA (CLCAE) and compare them with the chitosan-coated liposomes of asiatic acid (CLAA) for oral delivery to treat the initial phases of AD.

**Methods:** The solvent evaporation technique was used to develop CLCAE and CLAA, optimised with the experiment's design, and was further evaluated.

**Results:** Nuclear magnetic resonance (NMR) studies confirmed coating with chitosan. *Transmission electron microscopy* (TEM) and atomic force microscopy (AFM) indicated the successful formation of CLCAE and CLAA. Differential scanning colorimetry (DSC) confirmed the drug-phospholipid complex. Furthermore, the rate of *in vitro* release of CLCAE and CLAA was found to be 69.43±0.3 % and 85.3±0.3 %, respectively, in 24 h. *Ex vivo* permeation of CLCAE and CLAA was found to be 48±0.3 % and 78±0.3 %, respectively. In the Alcl3-induced AD model in rats, disease progression was confirmed by Y-maze, the preliminary histopathology evaluation showed significantly higher efficacy of the prepared liposomes (CLCAE and CLAA) compared to the *Centella asiatica* extract (CAE) and they were found to have equivalent efficacy to the standard drug (rivastigmine tartrate). The considerable increase in pharmacodynamic parameters in terms of neuronal count in the CLAA group indicated the protective role against Alcl3 toxicity and was also confirmed by assessing acetylcholine (Ach) levels. The pharmacokinetic study, such as C _max_, T _max_, and area under curve (AUC) parameters, proved an increase in AA bioavailability in the form of CLAA compared to the pure AA and CLCAE forms.

**Conclusion:** The preclinical study suggested that CLAA was found to have better stability and an ideal oral drug delivery system to treat AD.

## Introduction

**Figure 1.**
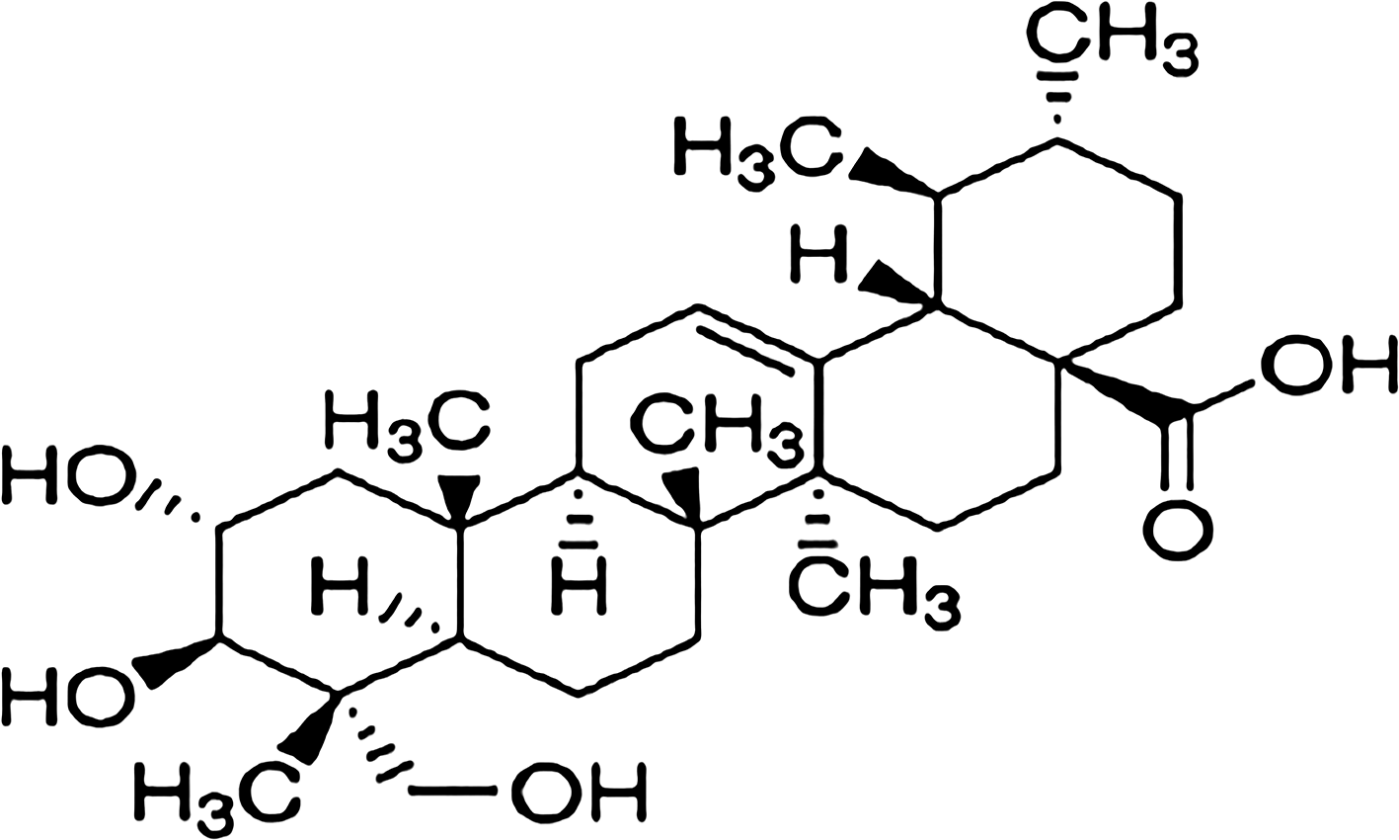
Structure of AA.

The most widespread form of dementia throughout the world is Alzheimer’s Disease (AD), which possibly involves environmental and biological factors leading to chronic, progressive neurodegeneration. ^1^ The ubiquity rate increases by approximately 1% to 40% at 60 to 90 years. ^2^ In the progressive stage of AD, amyloid plaques and tangled bundles of proteins are responsible for blocking neurotransmission signals, resulting in short-term memory loss, anterograde amnesia, frustration, and mood swings associated with neuropsychiatric problems. ^3^^,^^4^ Therefore, diagnosis and early stages of treatment, i.e., long-term and concomitant medication are essentially required to manage AD effectively. Currently, there is no specific medication available to cure AD completely. However, some studies have reported that progression can be delayed by treating patients with cholinesterase inhibitors. Cholinesterase inhibitors were shown to prevent the breakdown of Ach neurotransmitters responsible for memory and thinking. ^5^ The translation of natural medicinal treasures into a suitable form for preventing or curing a particular disease is emerging in various research domains. ^6^ The use of phytoconstituents in treating diseases is more promising due to their clinical potential, with a better safety margin and cost-effectiveness than synthetic alternatives. ^7^ *Centella asiatica* (CA) plants have the highest concentration of triterpene glycosides. Asiatic acid (AA) has acquired prominence among triterpenoids due to its various pharmacological activities against a variety of illnesses. ^8^ AA ( Figure 1) has been discovered as the most active chemical capable of rejuvenating neurons. It safeguards against the hippocampus' neurogenesis declining and memory deficits. ^9^^–^^11^

The oral route is the most suitable mode for long-term medication with its ease of administration, coupled with the highest degree of patient compliance and reasonable treatment persistence. ^12^ However, most phytoconstituents possess poor aqueous solubility, low permeability, and diffusibility, which affect their bioavailability. Similarly, poor solubility of AA results in poor absorption, leading to compromised bioavailability, thereby limiting its clinical use. The efficacy of any formulation is determined by the amount to which active ingredients are delivered to the target location at the required therapeutic concentration and exposure period. ^13^^,^^14^ Liposomes are nano-vesicular systems for targeted drug delivery that protect the drug from the external environment. This transport system can bypass the drug degradation in the liver, thus enhancing bioavailability. ^15^ Conventional liposomes for oral distribution are unsuitable because they degrade rapidly in the environment of gastric acid and other metabolic enzymes found in the gastrointestinal (GIT) system. Conventional liposomes can be modified by including polymers, macromolecules, polysaccharides, antibodies, or aptamers to advance the brain's targeted delivery and blood circulation time. ^16^ The chitosan-coated liposome is one such modification that improves oral drug delivery. Chitosan is a biodegradable polymer that contains d-β (1→4)-linked N-acetyl-D-glucosamine (A) and D-glucosamine (D) sugar units. Due to its ionic charged interlinkage between a negative charge phospholipid and a positive charge amino group, chitosan strengthens stability and prevents drug degradation from the lipid bilayer by creating a long-lasting biocompatible coating on the lipid membrane. Furthermore, the mucoadhesive characteristics of chitosan result in a prolonged residence period well at the location of absorption and extended release of the drug. ^17^ The oral efficacy of AA can be altered significantly by incorporating it into chitosan-coated liposomes. It is a promising approach to halt the progress of the disease in the early stages because of its convenient mode of administration with resistance to enzymatic destruction and exhibits sustained drug release. ^18^^,^^19^

Therefore, the work is intended to fabricate and compare chitosan-coated liposomes of *Centella asiatica* extract (CLCAE) and chitosan-coated liposomes of asiatic acid (CLAA) to promote the oral absorption and bioavailability in the treatment of the initial stages of AD.

## Methods

### Materials

97% pure asiatic acid and 75–85% deacetylated chitosan of 50kDa were acquired from Sigma-Aldrich, USA. *Centella asiatica* extract was prepared using the soxhlation method in the college laboratory. “Phospholipon® 90 G” was obtained as ex-gratis from Lipoid®, Germany. Rivastigmine was acquired from Yarrow Chem Products. Cholesterol, dichloromethane, dimethyl sulphoxide (DMSO), and methanol were purchased from Himedia Laboratory Private Limited. Chemicals & reagents like orthophsosphoric acid and acetonitrile were analytical or HPLC grade and did not require any extra purification.

### Lab animals

The NGSM Institute of Pharmaceutical Sciences' Institutional Animal Ethics Committee (IAEC) approved the purchase of adult Wistar rats (both male and female) weighing approximately 200–250 g, from the Nitte University-affiliated centre for animal research and experimentation (NUCARE) (Approval No -NGSMIPS/IAEC/MARCH-2018/88). In accordance with committee for the purpose of control and supervision experiments on animals (CPCSEA) rules, experiments were conducted. Six groups of six animals each were maintained in a cage, with the optimum conditions of 25°C, 50% RH, and 12-hour light/dark intervals with regular feedings including water and pellets food (Krishna valley Aggrotech, Sangli, Maharashtra, India).

### Collection, extraction, and standardisation of CA (CAE)

To maximize phytoconstituents, the CA plant was harvested early in the morning from a fertilized area during the rainy season. ^20^ The plant was authenticated, followed by preliminary evaluations. The CA plant was dried at 45°C for two weeks, powdered, and subjected to soxhlet extraction with the solvent methanol. The selection of the solvent was based on the phytochemical test. The CA extract was filtered, dried, and stored airtight for further studies. The amount of phytoconstituents, mainly AA, considered a standard drug for further investigations in the extract, was estimated using the RP-HPLC method.

### Formulation of conventional liposomes of CAE (LCAE) and AA (LAA)

The liposomes are prepared according to the modified solvent evaporation method. ^21^ AA, SPC (soy phosphatidylcholine) and cholesterol were mixed in various ratios with 25 mL of dichloromethane reagent in 100 mL round bottom flask. To acquire the film, it was attached to a rotary flash evaporator with a set 60 rpm speed at 60°C. Phosphate buffer pH 6.5 was taken to hydrate the film at ambient temperature. ^22^

### Design of experiments (DOE)

Design expert software was utilised to explore the outcome of various parameters to get an optimised formulation. The effect of parameters, such as the molar ratio of drug: lipid (X1) and the ratio of SPC: cholesterol (X2) as independent variables, on vesicle size (Y1) and entrapment efficiency (Y2) as dependent variables was investigated at three levels: low, middle and high concentration representing -1, 0 and +1 respectively. ^23^^,^^24^ The software Design Expert version 11.0.3.0;64-bits stat- Ease, Minneapolis; USA (RRID:SCR_002427) prepared and evaluated for the response suggested nine formulation combinations. The obtained results were analysed and validated using an analysis of variance (ANOVA)(RRID:SCR_002427) The following quadratic equation was taken to assess the implied model:

Y=b0+b1X1+b2X2+b12X1X2+b11X12+b22X22+.……… (1)

Where “Y” is the sum of calculated responses from each factor level, “b0” is an intercept. The regression coefficients “b1” to “b22” are calculated from the experimental values of “Y,” and “X1” and “X2” are the implied levels of independent variables. Table 1 displays experimentally obtained values of independent variables.

**Table 1.** 3 ^2^ level factorial design variables for optimised formulation: coded levels and actual values for each factor.

Factors	Levels, actual (coded)		
	-1 (Low)	0 (Medium)	+1 (High)
Independent variables			
A = Drug:SPC ratio (w:w)	1:5	1:10	1:15
B = SPC:Cholesterol ratio (w:w)	70:30	60:40	50:50
Dependent variables	Goal		
Y1 = Vesicle size (nm)	Minimise		
Y2 = Entrapment efficiency (%)	Maximise		

### 1H -NMR study (Proton nuclear magnetic resonance)

1H-NMR spectrometry is employed to quantify the drug's encapsulation in a bilayer lipid membrane. The 1H-NMR spectra of pure AA, SPC, and the optimised liposome formulation were taken and analysed for the carbon-hydrogen framework of every single component. ^25^ A specific quantity of samples was liquified in DMSO, filled into NMR tubes, and analysed by a 400 MHz Fourier transform nuclear magnetic resonance (FTNMR) spectrophotometer (Bruker Ascend, Rheinstatten, Germany).

### Formulation of chitosan-coated liposomes of CAE (CLCAE) and AA (CLAA)

The modified ionotropic gelation was used to design CLCAE and CLAA. ^26^ In 0.5% w/v acetic acid, chitosan was dispersed. The mixture was maintained at room temperature overnight. For one hour, the mixture was treated with dropwise optimised liposomal suspension while being continuously magnetically stirred at room temperature. Liposomes were left undisturbed for 3–4 hours to swell. An ultra-probe sonicator was used to sonicate liposomal suspension for 30 minutes to produce chitosan-coated liposomes. The prepared formulation was kept at 5°C in an airtight container for further studies. ^27^

### *In vitro* characterisation of CLCAE and CLAA

**Size of the vesicles, poly dispersity index (PDI), and zeta (ζ) potential**

The formed vesicle average size, PDI, and ζ-potential of optimised CLCAE and CLAA were observed by the Malvern zeta sizer, which operates based on the dynamic light scattering principles. Prior to analysis, sample dilution was made in the ratio of 1:10 with the ion-free water. To assess the physicochemical parameters and stability of the synthesised liposomes, the study was triple-checked at 25°C.

**% Entrapment efficiency (EE)**

A quantitative method was used to determine the % EE of the optimised CLCAE and CLAA. ^28^ A white pellet was formed by centrifuging the sample at 20,000 rpm for one hour at 4°C in a refrigerated centrifuge. The unentrapped drug was examined by separating the supernatant. The sample remaining in the basal portion of the tube was mixed with 0.1N 500 μl of sodium hydroxide and meticulously vortexed for three minutes. The Triton X-100 was added 5mL to make a clear colloidal mixture and further centrifuged for two more minutes to separate the drug from the enveloped vesicles.

The % of the entrapped drug was determined using RP-HPLC using the following formula,

$$\left(\%\right)\mathrm{EE}=\frac{\text{Drug in pellet}\;\left(\text{entrapped drug}\right)}{\text{Total drug added}\;}\times 100$$

### High-performance liquid chromatography

Hebbar *et al*. developed and validated the method of RP-HPLC technique for AA as per ICH harmonised tripartite guideline. ^29^ The stationary phase was column C-18 with 250 mm x 4.6mm x 5μ (Phenomenex Luna Omega) and the solvent system included 0.1% orthophosphoric acid and acetonitrile with 1 mL/min flow. Photo diode array was used as a detector at a wavelength of 210 nm. The calibration curve was plotted using a stock AA solution. The method's limit of detection and limit of quantitation were found to be 0.784507 μg/mL, and 2.615 μg/mL respectively. The regression equation was y=3790.1x-3001.9, 9.69.6±0.22 minutes was the retention time for the AA and 0.9987 was the correlation coefficient (r ^2^).

### Drug content analysis

The AA content in both liposomal formulations (CLCAE and CLAA) was determined through the RP-HPLC method as mentioned above. The formulations (1 mL each) were dispersed separately in 10 mL of methanol. The samples were membrane filtered and diluted appropriately to estimate the drug content.

### Liposomal stability test in simulated gastric fluid (SGF)

Measurement of turbidity determines the stability of CLCAE and CLAA as per the study reported by Chang-Moon Lee *et al*. ^30^ CLCAE, CLAA, and uncoated liposomes were added to SGF. The pH 1.2 SGF was made using 0.2% w/v sodium chloride, 0.7% v/v of hydrochloric acid, and 0.32% w/v pepsin. Turbidity and chitosan concentrations are interrelated; the more the turbidity, the more probable it is that chitosan will be lost from the vesicles. The turbidity of the liposomes was measured using a Digital Nephelo Turbidity Metre, and the results were represented in nephelometric turbidity units (NTU). ^31^

### DSC analysis (Differential scanning calorimetry)

The specific changeover temperature difference of the samples such as AA, SPC, its mixture and formulation CLAA were measured through DSC (Q20, TA Instruments USA). ^32^ The samples were exposed to the temperature 0 to 400°C at a heating rate of 10°C/min. The universal software version 4.5A, TA instruments USA was taken to quantify peak transition temperature.

### TEM analysis (Transmission electron microscopy)

The morphological characteristics such as the shape and appearance of CLCAE and CLAA were determined through TEM (version JEM-100s, JOEL, Japan). ^33^ The samples were diluted in the ratio 1:20 with the ion-free water before the analysis and further sonicated for 3 min. The sample droplet was individually deposited onto a metal plate made up of copper that had been coated with carbon to create a thin film. One drop of colouring pigment i.e., 2% w/w ammonium molybdate with 2% w/v ammonium acetate of pH 6.8 was added to the film. It was examined under a transverse electron microscope and compared both the sample surface morphology.

### AFM analysis (Atomic force microscopy)

The three-dimensional surface structure analysis of the formulation CLCAE and CLAA were carried out through AFM (Innova SPM, USA). ^34^ Each sample was placed separately as a smear and observed through the range of resonance frequency 267-329kHz with the scanning speed 1.2Hz of AFM tips.

### *In-vitro* drug release studies

The % drug release from CLCAE and CLAA were measured through Franz diffusion cell using sigma dialysis membrane and the release rate was compared with AA and CAE. 1 mL sample (100 mg/mL) was kept on one side, while the other part of the membrane was filled with the acidic buffer pH 1.2 (200 mL) dissolution media for two hours. It was then replaced with phosphate buffer pH 7.2 (2–24 hours) with 0.25% w/v sodium lauryl sulphate as two separate media to simulate the stomach and intestinal conditions. The media were sustained at the temperature of 37°C on a magnetic stirrer. A 5 mL sample was withdrawn with the specific intervals and the condition of the sink was kept constant by refilling the same volume of media. Samples were analysed and compared using RP-HPLC at 210 nm. To assess the drug release strategy from the optimised CLAA, the data were fitted to various *in vitro* kinetic release models like Korsmeyer-Peppas, Higuchi, Zero and First Order models. ^35^^,^^36^

### *In-vitro* antioxidant activity study

The prepared formulations' and AA's abilities to neutralise free radicals were determined *in vitro* and compared with standard ascorbic acid. In this method, free radical DPPH (2,2-diphenyl-1-picrylhydrazyl) was selected as described by Jamuna S. *et a*l. ^37^ 1.5 mL of 0.1mM free radial DPPH was treated with 3.5mL of 10 to 50 μg/mL concentrated methanolic solution of AA, CAE, optimised CLCAE, and CLAA. ^38^ The absorbance of all the samples was measured at 518 nm through ELISA plate reader (version AM-2100, USA). The percentage of DPPH inhibition was measured through the formula.

$$\%\text{Inhibition of DPPH}=\frac{\text{Absorption}\;\left(\text{control}\right)-\text{Absorption}\;\left(\text{sample}\right)}{\text{Absorption}\;\left(\text{control}\right)}\times 100$$

### *Ex vivo* drug permeation analysis

The drug intestinal permeation studies of both the optimised formulations were determined using non-everted gut sac procedure. ^39^^,^^40^ The rats were sacrificed and small intestine was treated with oxygenated saline. The intestine was split into 30.5 mm-diameter sacs that were 8 cm long and loaded with optimised CLCAE and CLAA (~100 mg AA). The thread was used to bind the sac's ends and located in a conical flask filled with ringer’s solution of pH 9 (10 ml). 37°C temperature was kept constant in a water bath shaker at 75 rpm with 5% CO _2_ aeration. Every 20 min, for a maximum of 8 h, the samples were removed and substituted with the fresh media. RP-HPLC was used to analyse the screened sample.

### *In vivo* evaluation of optimised CLCAE and CLAA and their protective role in AD

**Experimental design**

The animal experiments were carried out in the month of January (17/01/2019). The treatment (once a day, *p.o*) was administered for 89 days as described below:

1. Normal control: 0.9 % w/v NaCl, (5 ml/kg)
2. Disease control: AlCl _3_ (50 mg/kg)
3. Positive control: AlCl _3_ (50 mg/kg) + Rivastigmine (1 mg/kg)
4. AlCl _3_ (50 mg/kg) + CAE (5 g/kg)
5. AlCl _3_ (50 mg/kg) + CLCAE (100 mg/kg)
6. AlCl _3_ (50 mg/kg) + CLAA (100 mg/kg)

The behavioural assessment was performed using the Y-maze to evaluate disease induction on the 45 ^th^ day and 90 ^th^ day, followed by a histopathology study. The experiments were concluded on the 95 ^th^ day (21/04/2019) from the first day of the experiment. ^41^^–^^44^

### The mechanism of action of the AlCl _3_ model

Aluminium ions are a potential neurotoxic agent. This trivalent cation binds to IRP (iron regulatory protein) and stimulates AβPP as well as ferritin. APP's inappropriate overexpression will result in more Aβ being produced (which is resistant to protease enzyme because Al ion associated), accumulates mainly in the hippocampus region. By promoting tau phosphorylation and iron-induced lipid peroxidation, Al ions contribute to neurodegenerative processes. A change in the concentration of free iron ions, the production of free radicals, and the aberrant expression of ferritin all contributed to oxidative damage and membrane lipid peroxidation. In AD, these occurrences ultimately result in neuronal death. ^45^

### Behavioural parameters: Y-maze

The Y-maze model was used to determine the spatial working memory and assess rodents’ continuous altering behaviour. In this method, the animals were involved in a suitable search operation using food as a reward. The animals were trained prior to the commencement of the experiment. ^46^ The Y-maze consisted of three horizontal arms that were allied at an angle of 120°. The maze arms had walls of 40 cm in length, 3 cm in width, and 12 cm in height. The three arms were labelled as the start arm in which the animal started to explore (A), a reward arm containing food stimuli (B), and another random arm (C). The maze was made up of dark polyvinyl plastic that was opaque. For the test, the animals were put in the start arm and given free rein to explore the maze. The sequence of each arm and entry was recorded for 8 min. The arm entry was tabulated; only alternated arm records such as ABA, BCB, CBC, and so on—rather than repeated arm entries were taken into consideration during the trial. The arm entries and the alternations were recorded, and % alternations were measured.

$$\%\text{Alternation}=\frac{\text{Number of alterations}}{\text{Total}\;\mathrm{arm}\;\text{entries}-2}\times 100$$

The locomotor activity of the animals was estimated with the help of the arm entries taken place. ^47^ All experiments were carried out under standard laboratory conditions.

### Histopathology of the brain

The rats were euthanized on the 90 ^th^ day. The brain was perfused using ice-cold normal saline and then dissected to isolate the frontal cortex and hippocampus. Hippocampus was sectioned, and tissue processing was done. The tissue was stained with 0.1 % crystal violet stain and observed under the microscope (Zeiss Primo Star Digital, Germany). Photographs of the CA1 and CA3 areas of the hippocampus were taken, and the number of normal healthy neurons out of 100 neurons was counted with the help of image J software (version 1.53t). ^48^

### AChE (Acetylcholinesterase) assessment study

Acetylcholinesterase (AChE) is primarily a major enzyme in the cholinergic system. AchE was estimated by the Ellman method. ^49^ To the rat brain tissue homogenate (100 μl), 0.1 M phosphate buffer pH 8 (650 μl) and DTNB (Ellman reagent) (0.1 mL) were blended after being added. The mixture was treated with 0.1 mL acetylthiocholine iodide, and a UV-visible spectrophotometer at 412 nm, the absorbance was determined (UV-1800, Shimadzu, Japan). The brain tissue homogenate was substituted with 100 μL of purified water to make the blank.

### The oral bioavailability study

**Estimation of AA in rat serum**

The sum of drug availability after oral intake of AA, CLCAE, and CLAA was performed on three groups of 150-200g wistar rats (n=6). Prior to the procedure, the animals underwent an overnight fast with unlimited access to water. Group I was treated with a single dose of AA (100 mg/kg,p.o.) whereas group II, and III animals received single dose of optimised CLCAE and CLAA (~100 mg/k.g AA, p.o.), respectively. After the dosing, the animals were partially anesthetised using either, from the retro-orbital plexus 1 mL of blood samples were collected in centrifuge tubes at a predetermined time of up to 8 h. To isolate the contents, the serum sample was spun up at 3000 rpm for 10 min. The AA was quantified by RP-HPLC. ^50^

**Extraction of AA from plasma**

Initially, the mixture of 1 mL serum and 5 mL methanol were taken in a volumetric flask at room temperature. The sample was properly mixed before being heated for 30 min at 55°C. After that, a 10 mL flask was made up with methanol and centrifugation for 30 min at 5000 rpm (Remi Elektrotechnik Ltd. India). 20 μL of filtered supernatant was taken for analysis.

**Pharmacokinetic measurements**

Depends on plasma concentration-time curve parameters, the C _max_ and T _max_ of CLCAE and CLAA were analysed. The pharmacokinetic parameters were successfully interpreted through PK/PD computer software version 4.1, USA, which includes measurement drug plasma concentration from zero to last measured sample (AUC _0 -t_) and from zero to infinity (AUC _0-∞_) measurement of drug elimination half-life (K _1/2el_) and elimination rate constant (K _el_) clearance (Cl) and volume of distribution (V _d_). The relative bioavailability (F) of both the formulations was determined using the following formula. ^51^

F=Total amount of drug absorbed from formulation(Amaxformulation)Total amount of drug absorbed from pureAAAmaxpureAA×100

$$F=\frac{\left(V_{d}\;X\;K_{\mathrm{el}}\;X\;\mathrm{AU}C_{0-\infty }\right)\text{formulation}}{\left(V_{d}\;X\;K_{\mathrm{el}}\;X\;\mathrm{AU}C_{0-\infty }\right)\;\text{pure}\;\mathrm{AA}}\times 100$$

The obtained data of behavioural parameters and pharmacokinetic studies were stated as mean±standard error mean (SEM). Student t-tests and one-way analysis of variance (ANOVA) were used for the statistical analyses. Statistical were regarded for P values <0.05.

### Stability study

The formulated optimised CLCAE and CLAA underwent a stability investigation for three months at two distinct settings, refrigeration and room temperature (5 ±2°C), (32 ±2°C) at 60 ±2% RH, respectively. Up to three months, each month samples were taken, diluted appropriately with 7.4 pH buffer for determine EE, vesicle size and zeta potential ^52^

## Results and Discussion

### Extraction and standardisation of CAE

CA plants that were harvested, were primarily investigated for quality criteria. The safety of its consumption was determined by total ash value, insoluble acid ash value, and water-soluble ash value, which were found to be 16±0.23%, 3±0.21%, and 3±0.43%, respectively. CA showed the total moisture content of 7.2 ± 0.62%, signifying better stability. The methanol solvent in the soxhlet extraction process yields maximum phytoconstituents compared to solvents like, chloroform, petroleum ether, n-butanol, ethyl acetate and solvents screened by chemical tests. Methanol's amphiphilic nature allows it to dissolve almost all compounds irrespective of the polarity. The obtained extract was standardised and its terpenoids estimated using the RP-HPLC method. AA was considered a reference compound for subsequent drug quantification; with a retention time of 15.79 min, the compound AA was identified, which was found to have approximately 10% availability in the total extract.

### Formulation of conventional liposomes of CAE (CLCAE) and AA (CLAA)

The pre-formulation study was conducted to improve the solubility of AA. AA is a BCS IV drug that has poor aqueous solubility and permeability. Therefore, organic solvents were used in the initial screening. A clear solution was formed in dichloromethane that also possessed a reduced boiling point (39.6°C) and reduced toxicity (LD50 value of 1.2 g/kg in rat *p.o*). Therefore, to prepare the formulations, dichloromethane was chosen as a solvent. The drug: SPC ratio was set at 1:5 to 1:15 based on the literature research, and the SPC: cholesterol ratio was set at 70:30–50:50 based on the DoE. A rotary flash evaporator was used to evaporate mixtures that had been liquified in dichloromethane solvent. The film was dried for nearly 45 min at 45°C using a vacuum pump before hydration to remove the organic solvent. Because liposomes are prone to fusion and drug leakage, to enhance its rigidity and stability, cholesterol was added. ^53^^,^^54^ Using an ultra-probe sonicator, the resulting multilamellar vesicles (MLVs) were pulverised to reach the required size range of less than 250 nm. Furthermore, an investigation was conducted to study the factors that influence the formulations. ^55^

### Design of experiments (DOE)

Table 2 displays the results derived using a three-level multifactorial randomised polynomial equation model for the two dependent factors for the range of formulations according to the study design. ^56^ The obtained values from the experimental trials exhibited a considerable variation in vesicle size (142-277 nm) and % EE (56.08%-82.38%). The model's as well as its parameters' relevance generated, were analysed by ANOVA. The conclusions were made based on polynomial equations ( equations 2 and 3). The positive sign before the factors indicated that the response and the factor had a linear relationship, the negative sign, on the other hand, denoted inverse correlation between both.

$$\text{Vesicle size}=+226.56+42.50^{*}A-20.17^{*}B$$ (2)

$$\%\mathrm{EE}=+72.54+7.61^{*}A+4.64^{*}B$$ (3)

**Table 2.** 3 ^2^ level factorial randomised quadratic design experimental trial batches, with obtained vesicle size (nm) and entrapment efficiency (%).

		Factor 1	Factor 2	Response 1	Response 2
Std	Run	A:Drug:SPC	B:SPC:Cholesterol	Vesicle Size	Entrapment efficiency
		w:w	w:w	nm	%
1	1	-1	-1	219	56.08
9	2	1	1	246	82.38
4	3	-1	0	161	67.4
8	4	0	1	214	77.13
6	5	1	0	276	80.24
5	6	0	0	277	75.51
3	7	1	-1	255	75.76
2	8	0	-1	249	69.08
7	9	-1	1	142	69.24

A and B the implied values for the AA: SPC and SPC: cholesterol ratios, correspondingly. The model developed for vesicle size was significant since it had a p-value of 0.05 and an F-value of 7.87. The difference between the adjusted R-squared value (R ^2^ = 0.6318) and the predicted R-squared value (R ^2^ = 0.5257) was less than 0.2, indicating that the models agreed rather well. As shown in equation 1, the AA: SPC ratio had a prominent effect on vesicle size. The model developed for entrapment efficiency was significant, with a p-value of 0.05 and an F-value of 35.72. The difference between the adjusted (R2 0.8967) and predicted (R2 0.8080) below 0.2 R-squared value indicated good correlation. As shown in equation 2, the AA: SPC ratio and the SPC: cholesterol ratio significantly affected the entrapment efficiency. The prepared liposomes were found to have a vesicle size of 140 to 280 nm and a % entrapment efficiency of 50% to 80%. The optimum concentration was selected based on desirability values ( Table 3). The vesicle size of the optimised liposome was 209.8 nm, and the percent entrapment efficiency was 71.2 ±0.03 %.

**Table 3.** Selected solution and the % error between the predicted and the observed values.

Factors		Responses	
A: Drug:SPC (w:w)	B: SPC: Cholesterol (%w/w)	Vesicle size (nm)	Drug entrapment efficiency (%)
Predicted			
1:6.88	50:50	212.86	72.40
Actual			
		209.8	71.27
% Error:		1.40	1.45

### 1H-NMR Study

**Figure 2.**
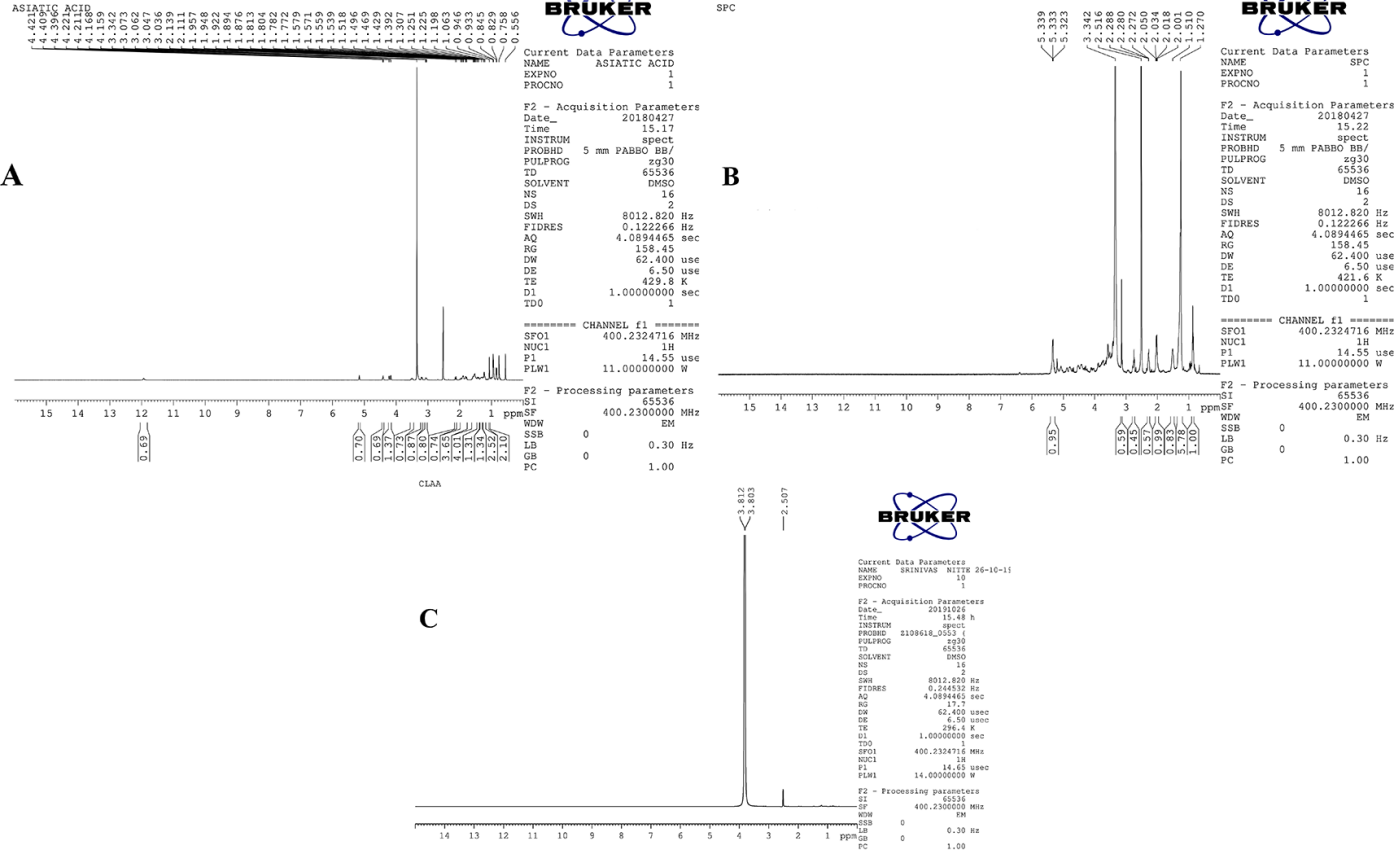
NMR of A) AA, B) SPC, C) Optimised liposome.

The 1H-NMR spectrum of pure AA is in Figure 2A, which shows different types of protons and its allocation corresponds to the type of hydrogen in the full structure of AA. The SPC 1H-NMR spectra are in Figure 2B. Basic chemical displacement values were observed. The chemical shifts of pure AA and optimised liposomes of AA were examined, with the downfield aromatic region (>7) and the upfield aromatic region, ^4^ showing most significant differences. The alkyl side chain of the optimised liposome was observed at δ 2.50, and the N-methyl groups corresponded to the chemical shift at δ 3.81. The alteration in proton signs in the aromatic area clearly demonstrated the establishment of molecular bonds with AA. The molecular interface was indicated by the weak intermolecular interaction between the phospholipid mixture and the phenolic region of AA. This confirms the formation of bilayer vesicles with AA. ^57^

### Formulation of chitosan-coated liposomes of CAE (CLCAE) and AA (CLAA)

In order to strengthen the mucoadhesiveness to the negatively charged cell membrane, chitosan was coated on their surface. This enhanced colloidal stability and regulated release. ^58^ It has been discovered that covering the surface of negative charges liposomes with chitosan is made easier by ionic exchanges among both the negative charge lipid group with the positively charged amino group of chitosan. ^59^ Drug leakage from the vesicular structure is also prevented by chitosan coating. ^60^

### *In vitro* evaluation of optimised CLCAE and CLAA

**Vesical size, PDI, and zeta (ζ) potential**

The influence of the structural integrity of the liposomes in a media is determined by vesicle size, PDI, and zeta potential. ^61^ When compared to conventional liposomes, chitosan covering increases the width and thickness of the vesicular system. Sonicated optimised CLCAE and CLAA have typical vesicle sizes of 224.4 nm and 209.8 nm, respectively., which signifies the optimum complex formation in which drug molecules tangibly bond with the lipid's polar heads to reduce the negative surface charge and the vesicle size. It plays a role in enhancing AA's absorption efficiency and sustained-release action. Optimised CLCAE and CLAA exhibited PDI values of 0.457 and 0.493, revealing that vesicle size distribution is in a narrow range. ^62^ **ζ** potential measurement is an essential tool to evaluate the surface electrical charge that indicates the potential stability of a vesicular system. Surface charges ranging from -20 to +20 mV have been found as a feature of ζ potential affinity accompanied by a stronger coagulation process than repellent force. ^63^ The phosphate group has a negative charge in the presence of water. The chitosan coat, on the other hand, contains more cationic polymers adsorbed to the surface of the liposome, resulting in a small positive charge. Optimised formulations CLCAE and CLAA displayed ζ potentials of 22.3 mV and 20.8 mV, respectively. The positive ζ charge of optimised CLAA shows good physical stability that ultimately favours the mucoadhesion property of the cell membrane and penetrates the mucous membrane.

**% EE**

By breaking the optimised liposomes using Triton X-100, the % EE was determined. The overall entrapment of AA in optimised CLCAE and CLAA was found to be 51.3 ± 0.03% (n = 3) and 71.2 ± 0.1% (n = 3 *)*, respectively. The results of the various studies on liposomal drug delivery and comparison with each formulation were satisfactory. Due to AA's limited water solubility and lipophilic character, EE has a higher propensity to entrap AA in liposome lipid bilayers due to its persistent vesicular shape. After an acid treatment or dialysis method, the unentrapped drug can be removed by centrifugation. To avoid drug loss in this study, the formulation preserved the unentrapped drug.

### Drug content analysis

Methanol was selected as a solvent because AA is insoluble in water. The standard calibration curve was plotted under a 10–50 g/ml linearity range. The regression equation was Y=3790.1x. The correlation coefficient (r ^2^) was 0.999 and the retention time was found to be 9.6±0.22 min. In the optimised CLCAE and CLAA formulations, the AA content was 43 ±0.02% w/w and 68±0.04% w/w, respectively.

### Stability study in simulated gastric fluid (SGF)

In the oral delivery system, the constancy of liposomes in the gastric fluid is a significant consideration. To confirm the stability of CLCAE and CLAA in comparison to uncoated liposomes, an *in vitro* stability test was performed. CLCAE and CLAA exhibited turbidity value of 11.2±0.004 NTU and 10.3±0.003 NTU, respectively. In contrast, uncoated liposomes showed 18.5±0.001 NTU, which confirmed the leakage of vesicles. This study indicated the stability of CLCAE and CLAA in SGF. The results of the current parameter indirectly support the better mucoadhesive by the electrostatic interaction of the chitosan-coated formulations. ^64^

### DSC analysis

**Figure 3.**
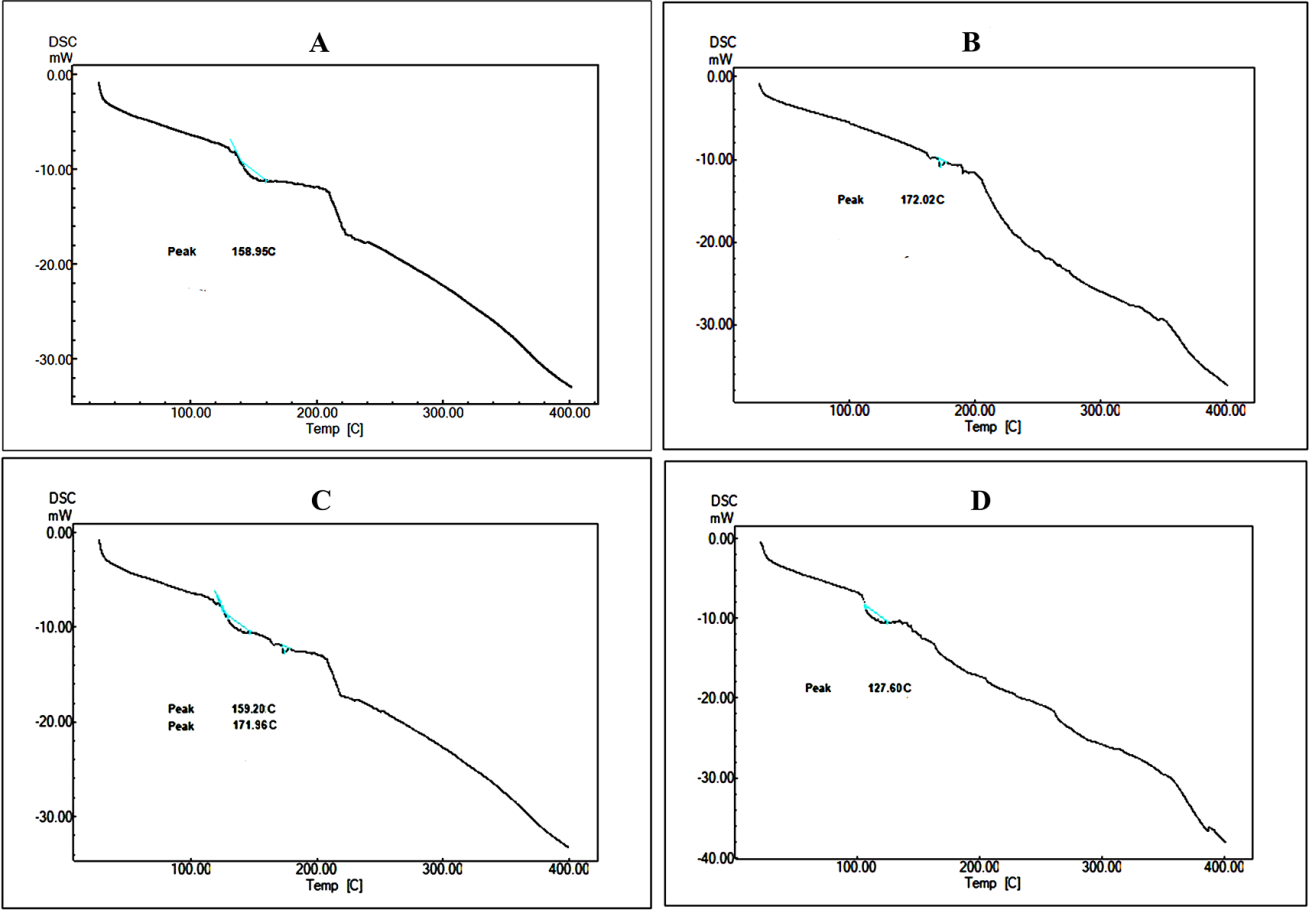
DSC thermogram of A) AA, B) SPC. C) PM D) CLAA.

DSC is a well-known method for investigating the thermal behaviour to describe complicated solid-state matter. ^65^ Purified AA exhibited a broad endothermic peak at 158.9 °C, which was in line with its melting point, as shown in Figure 3A. At 172.02°C, the SPC exhibited an endothermal peak ( Figure 3B), indicating that the physical state shifted from a gel to liquid form. The phospholipid's carbon group could have resulted in a different atom arrangement or crystal modifications. Two peaks were identified for the physical mixture of SPC and AA at 171.96°C and 159.02°C, respectively, matching to the peaks of SPC and AA. Furthermore, the optimised CLAA thermogram revealed a broad endothermal peak at 127.6°C ( Figure 3D). The fact that the peak shifted to lower temperatures could be attributable to the drug's improved solubility and lower crystallinity in the formulation form. It could be owing to hydrogen bonding or van der Waals forces combining AA with the tail of phospholipid molecules, resulting in the drug lipid complex. ^66^^,^^67^

### Surface morphology

**TEM**

**Figure 4.**
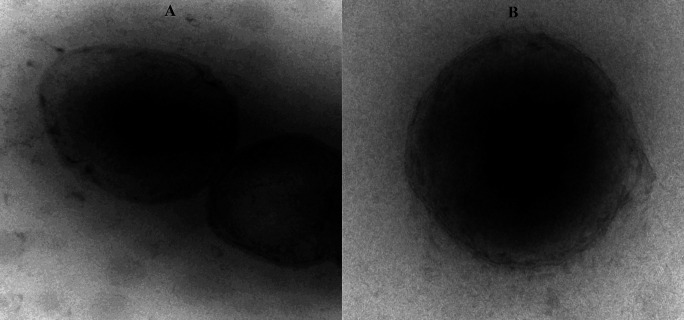
TEM of CLCAE and CLAA.

TEM images of both optimised CLCAE and CLAA ( Figure 4 (A, B)) were well-developed, detached, without any accumulation of vesicles, with particle size in the range of 196 nm and 187 nm, respectively. The obtained particle size of fabricated nanoconstructs can be considered as an ideal particle size for crossing blood brain barrier and bypassing reticulo-endothelial system (RES). ^68^

**AFM**

**Figure 5.**
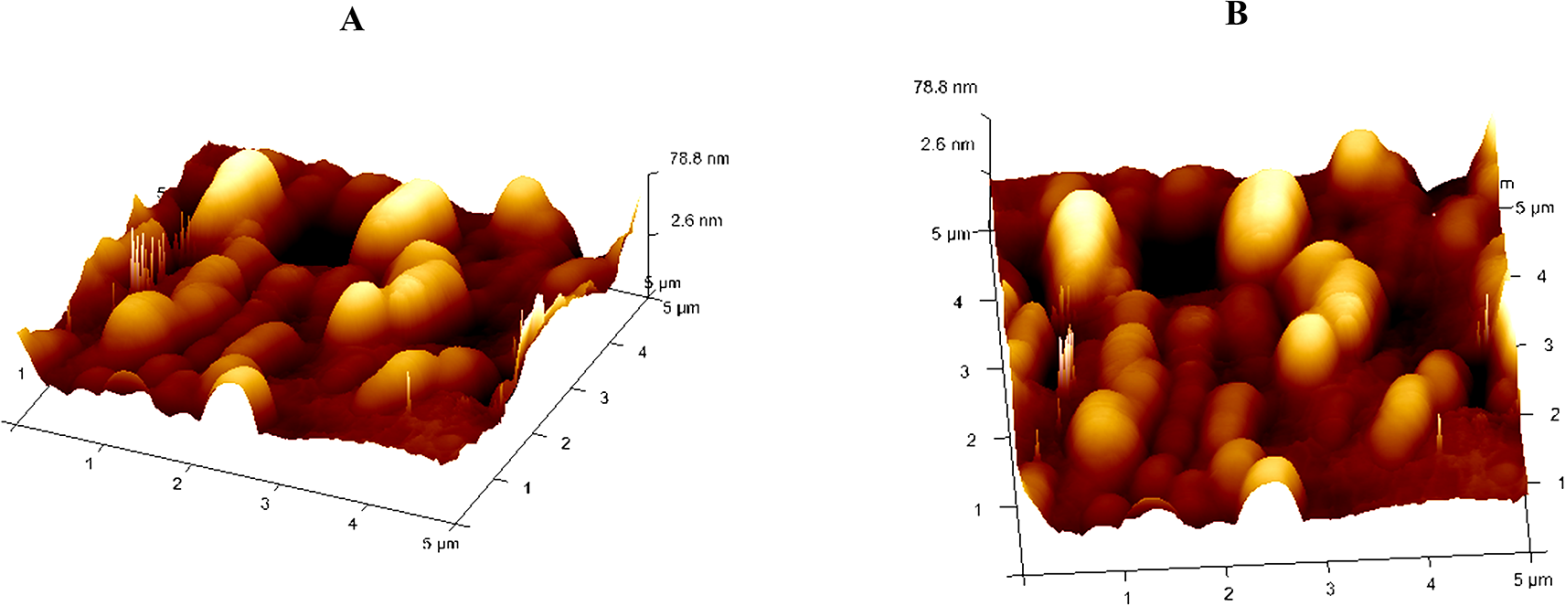
AFM of CLCAE and CLAA.

The distinct, well-formed spheres are revealed to be free of any agglomeration or degrading signs shown in the AFM image of optimised CLAA ( Figure 5B) and it might be the reason for the improved dissolution profile compared with the pure drug. However, the optimised CLCAE in ( Figure 5A) shows a vesicular nanostructure with a small aggregation and non-uniformity; this may be due to the vesicles' leakage, indicating less stability. ^69^

### *In vitro* drug release study

**Figure 6.**
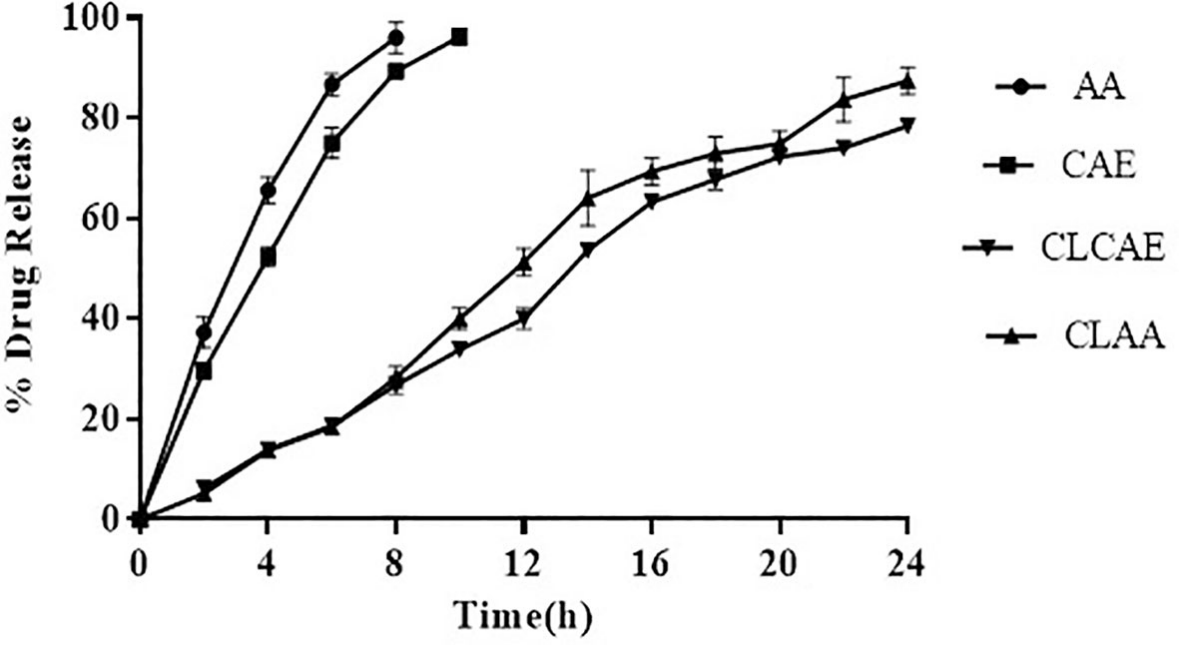
*In vitro* drug release of AA, CAE, CLCAE, CLAA (n=3).

The rate of drug release from the formulation CLCAE, CLAA, and pure form of AA would be useful to correlate *in vivo* drug release rate. In around 10 hours, AA exhibited a fast release of 65.34 0.30%. For AA, there was a further rapid decrease in drug release that persisted up to 16 hours. In optimised CLCAE, the sustained-release pattern was found, with CLAA showing 69.43% and 85.3 0.3% release in 24 hours, respectively ( Figure 6). The enhanced and sustained drug release from optimised CLAA is caused by the physicochemical alteration and electrostatic interactions of AA with SPC and chitosan. It presumably improved the complex's solubility along with wettability when compared to pure AA. ^70^ Kinetics of drug release of optimised CLAA was examined. Higuchi's plot of the optimised CLAA was linear with an R2 value of 0.972 and verified diffusion regulated drug release based on Fick's law. Korsmeyer-Peppas model showed n value being less than 0.4 (i.e., n = 0.3917), indicating quasi-fickian model. It depicts the drug layer's partial diffusion pattern. ^71^ The Higuchi plot was revealed to be the model that fit the data the best due to diffusion mechanism involved.

### *In vitro* antioxidant activity study

**Figure 7.**
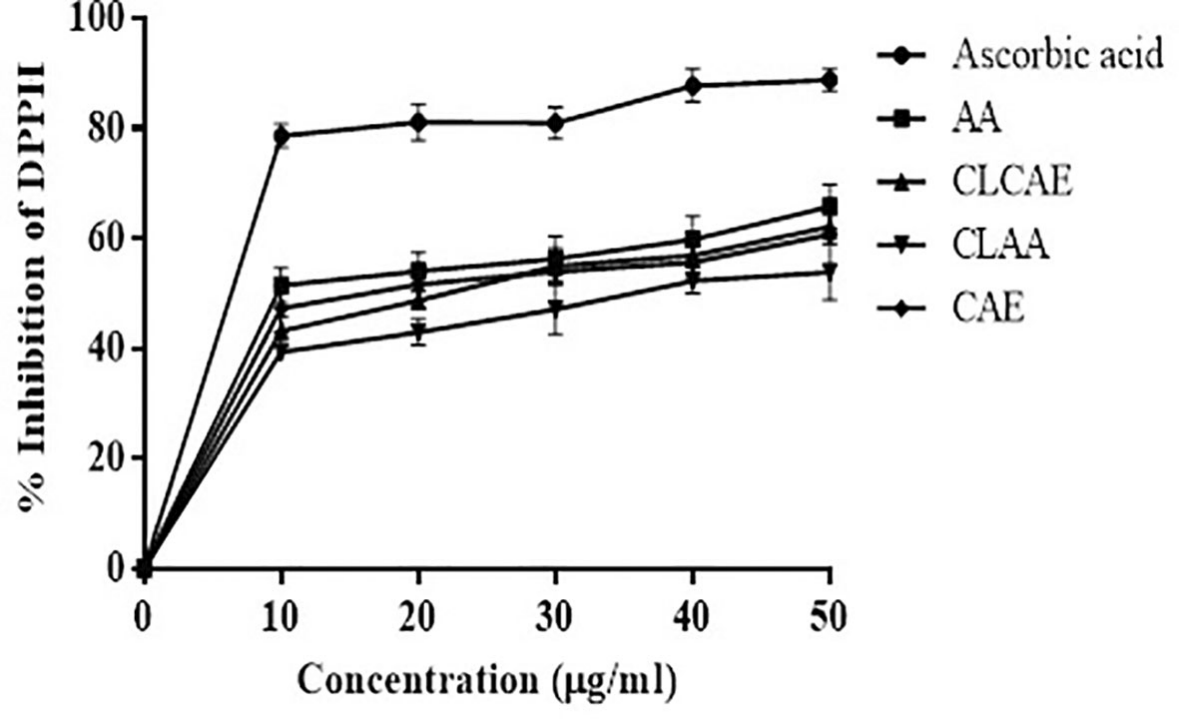
*In vitro* antioxidant study of CLCAE and CLAA (n=3).

The inhibition percentage of DPPH by AA, optimised CLCAE, and CLAA was compared to the reference ascorbic acid by taking equal concentration. At 50 μg/mL, the percentage of inhibition of DPPH for ascorbic acid, AA, optimised CLCAE, and CLAA, was found to be 90.75 ± 1.45%, 65.4 ±1.2%, 60 ± 1.3%, and 59.84 ± 1.6%, respectively ( Figure 7) (n ≥ 3). The results indicated that the pure drug exhibited substantial free radical scavenging activity and was also observed in liposomal formulations. It could be related to the AA's increased capacity to give hydrogen ions and to convert DPPH radicals to hydrazine equivalents. ^69^ Even after interacting it with the chitosan-coated phospholipid layer, AA's free radical scavenging action was intact.

### *Ex vivo* permeation study

**Figure 8.**
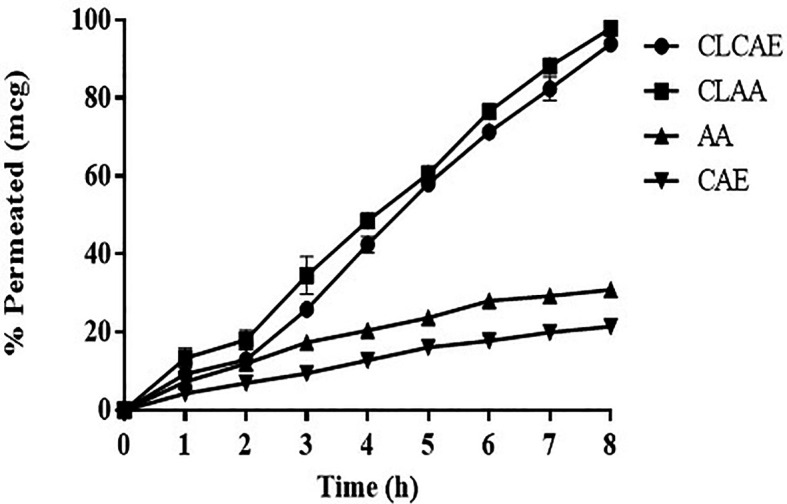
*Ex vivo* permeation study of AA, CAE, CLCAE and CLAA (n=3).

Using everted and non-everted intestinal sac models, it is possible to quantify the drug reception in the intestinal area. ^72^ Additionally, the non-everted sac models have a number of noteworthy advantages, including lesser volume of the sample and minimum structural damage to the intestine. The transport mechanisms were evaluated, and their relationship to *in vivo* drug absorption was correlated, using an *ex vivo* permeation analysis. In this study, the % permeation rate of optimised CLCAE and CLAA was analysed ( Figure 8) (n ≥3). The amount of drug permeated throughout 8 hours by optimised CLAA was better (97.9 ± 4.3%) than the optimised CLCAE (93.89 ± 4.03%) and showed a significant penetration rate difference. It most likely happened as a result of the chitosan's bio-adhesive ability. which causes a higher retention period and a prolonged adsorption rate in the mucosal region. The pure AA and CAE suspension demonstrated lesser permeation i.e., 30.90% ± 0.9% and 21.49% ± 0.76%, respectively *via* intestinal barrier as compared to CLAA and CLCAE. Thus, the results revealed that CAE and AA when incorporated in the nanocarrier may enhance its permeability *via* intestinal gut lining. Additionally, there was no substantial change in the % permeation between AA and CAE.

### *In vivo* evaluation of optimised CLCAE and CLAA and anti-AD studies

**Figure 9.**
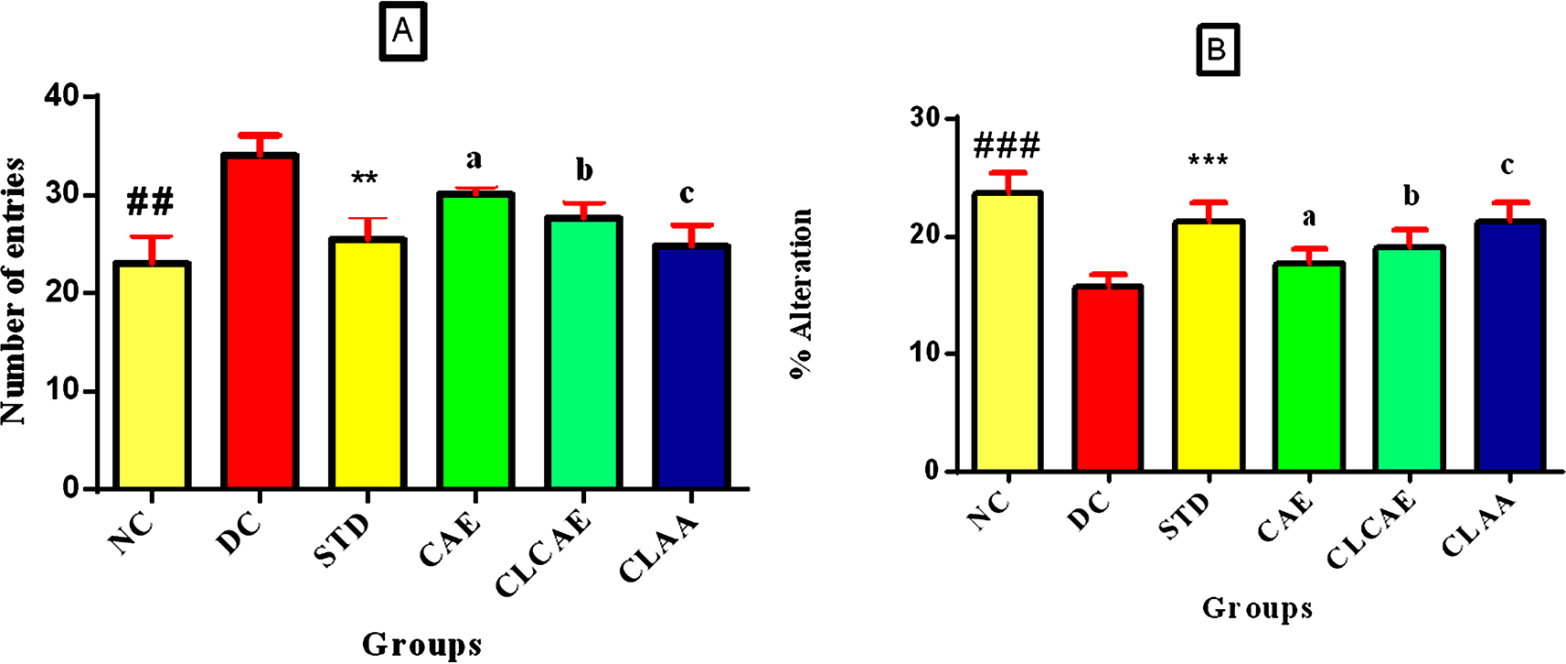
Y-Maze test. Note (bottom) Figure 9A: Data represented as mean ± SD. Statistical analysis was performed one way ANOVA followed by Bonferroni multiple comparison tests. ## *p< 0.05*, ** *p< 0.05,* b *p < 0.01, c< 0.01* when compared to disease control. Note (bottom) Figure 9B: Data represented as mean ± SD. Statistical analysis was performed one way ANOVA followed by Bonferroni multiple comparison tests. *## p< 0.01*, ** *p< 0.05, b p < 0.01, c< 0.01* when compared to disease control.

The wistar rat model was chosen for neurological investigations because it is easier to deal with, has a larger brain than the transgenic mice, and is less sensitive to human handling. Rats have relevant gene sequences (AβPP) for AD that are similar to human sequences (96.6%), merely three Aβ sequence differences. The Y-maze model was used to evaluate the effects of formulations on spatial working memory based on two parameters, i.e., the number of arm entries and the percentage alternations. ^73^ The memory retention activity of groups III, IV, V, and VI in terms of the number of arm entries by rats was carried out. Figure 9A shows groups V and VI (dose of ~100 mg/kg AA) exhibited a considerable drop in the sum of arm entries equated to group I. When compared to group I, group II exposed a substantial (p<0.05) increase in the number of arm entries, indicating a disturbance in memory and learning. Group IV (dose of 5 g/kg) showed fewer arm entries than group II. One-way ANOVA was used in the statistical analysis, followed by Bonferroni multiple comparison tests. The samples showed a dose-dependent and significant (p<0.001) rise in the percentage alterations compared to group I. When compared to group I, Group II showed a significant (p<0.001) decrease in percent alterations, which represents a disturbance in memory and learning. Figure 9B shows groups V and VI exhibited a significant (p<0.001) rise in the percent alteration when compared with group I. CAE, CLCAE, and optimised CLAA were found to preserve memory and learning in treated rats even after induction of AD. The optimised CLAA showed more promising results (p<0.05) than the CAE and CLCAE, which could be due to the greater bioavailability of AA.

### Histopathology

**Figure 10.**
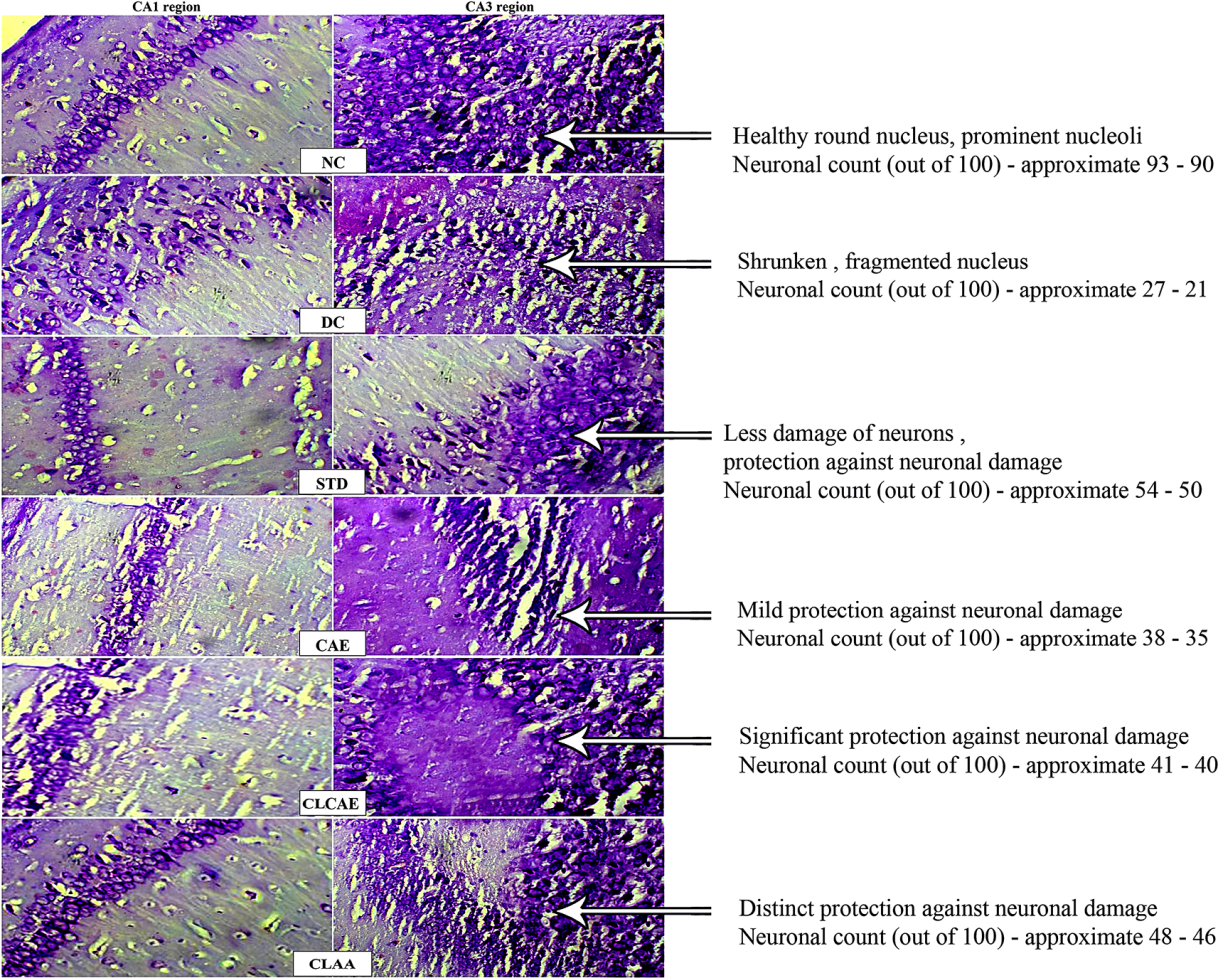
Histopathology study of A) Normal control, B) Disease control, C) Standard, D) CAE, E) CLCAE F) CLAA.

The processed brains of experimental rats were stained with crystal violet and eosin and examined by optical microscopy ( Figure 10). Group I revealed the presence of maximum neurons in cornu ammonis CA1 and CA3 regions and distinguished layers associated with the small blood vessels. The layers such as a plexiform layer, external and inner granular layer, and polymorphic cells were found. Besides this, the dentate gyrus was found to be healthy with a pale, and a round nucleus, and also well-defined nuclear boundary and prominent nucleoli were found. ^74^ Group II exhibited a massive cellular degeneration in the hippocampal region followed by neurofibrillary depletion. Dentate gyrus was found to be damaged darkly (basophilic) stained, with a shrunken and fragmented nucleus. The accumulation of aluminium in these regions leads to the formation of amyloid proteins. ^75^^–^^77^ In group III, loss and damage of the neurons were found in the CA3 area. However, not much damage and loss of neurons were found in the CA1 area. In this group, protection over neurodegeneration was observed. Group IV showed significant protection of neurons compared to group II. Group V and group VI showed loss of neurons in the CA1 region and dentate gyrus areas distinct protection compared to group II animals.

### Neuronal count

The CA1 and CA3 regions of the hippocampus sector are exposed to AD-type neurofibrillary degeneration. ^77^ The principal reason for a diminution of a neuron is the accumulation of amyloid plaques formed by the enzymatic breakdown of amyloid precursor protein (APP) and neurofibrillary tangles that is occurred by hyper-phosphorylation and oligomerisation of tau in this region. Consequently, it leads to disruption in neuronal transmission due to the slowdown of enzymatic signalling and nutrient supply to the neurons resulting in a decrease in the count of neurons. ^78^^,^^79^ In this study, the neuronal count found in CA1 and CA3 regions (out of 100) is given in Table 4. A considerable increase in the neuronal numbers was observed in groups IV, V, and VI compared to group II. However, neuronal numbers indicate a substantial protective role of formulated CLAA compared to CAE and CLCAE.

**Table 4.** Neuronal count (out of 100) in CA1 and CA3 region.

Groups	Neuronal count (out of 100)	
	CA1	CA3
Normal control	93.33±1.33	90.64±2.54
Disease control	27.58±2.41	21.34±3.29
Standard	50.75±3.70	54.21±1.47
CAE	35.83±2.30	38.67±1.40
CLCAE	40.34±7.34	41.10±1.36
CLAA	46.08±3.10	48.38±0.53

All the data were expressed as mean ±SE where n=6

### AChE assessment study

**Figure 11.**
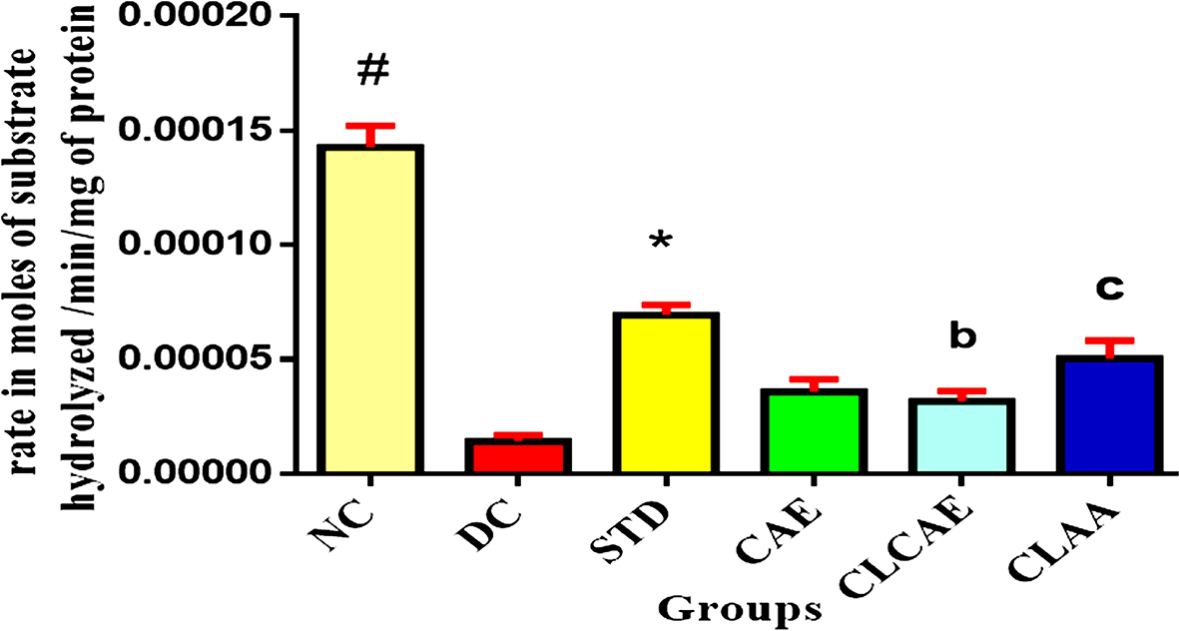
AChE assessment study of CLCAE, CLAA. Note (bottom): Data represented as mean ± SD. Statistical analysis was performed one way ANOVA followed by Bonferroni multiple comparison tests. ##p< 0.001, * *p< 0.01, b p < 0.1, c< 0.05* when compared to disease control *(n=6*).

AChE enzyme is responsible for the degradation of acetylcholine levels at a synaptic cleft region and influences the cholinergic neurotransmission. A single molecule of AchE can break down 5000 Ach molecules per second. ^80^ In Figure 11, (n=6) normal control (group I) shows a significant increase in the AChE level in the brain, and the level of acetylcholine found decreased as compared to group I (p< 0.05). The accumulation of AChE in the normal control group forms a network with Aβ peptide and stimulates amyloid fibril formation in the hippocampus region. ^81^ However, the positive control group showed substantial enhancement in acetylcholine levels by altering the active sites of AChE and inhibiting its activity. Group IV, V, and VI exhibited a significantly decreased AChE activity (p<0.05) and were found to have increased acetylcholine levels compared to group II. Furthermore, the group VI CLAA treated showed enhanced acetylcholine levels (p<0.05) compared to CAE treated group IV due to better bioavailability in the serum. The experimental facts herein revealed, a significant level of acetylcholine observed indicating that CLAA alters the enzymatic reaction of AChE and prevents the breakdown of Ach, which leads to the role of neuroprotection. ^82^

### Oral bioavailability study

The amount of drug that reaches into systemic circulations and the amount of drug absorption was determined. Herein, the oral bioavailability of optimised CLAA was compared with optimised CLCAE and AA ( Table 5). The *in vivo* oral bioavailability of CLAA in the initial hours was less in the serum due to the packed chitosan coat and later exhibited better bioavailability in the serum that lasted up to 8 hours. AA showed a maximum concentration in the first four hours. The maximum serum concentration of AA was found to be 3.43±0.12 μg/ml at 4 hours, and the optimised CLCAE was found to be 5.32± 0.34 at 6 hours. The optimised CLAA achieved 9.23±0.34 μg/ml of serum concentration at 6 hours and was sustained for an extended period of time. ^83^ It extended gut residence time by adhering to the intestinal mucosal layer by electrostatic interaction i.e., forming of a disulphide bridge between positively charged polymers with negatively charged cysteine-rich subdomains of mucus glycoproteins. It was found compared to the free drug that mucoadhesion enhances the portion of liposomal drug to passive permeation across the apical pole surface of intestinal epithelial. ^84^ The formulation CLAA showed substantial improvement (p< 0.05) in oral bioavailability with pure AA and CLCAE.

**Table 5.** Oral bioavailability study: Concentration of AA in rat serum.

Pharmacokinetic parameter	AA	CLCAE	CLAA
C _max_ (μg ml ^−1^)	3.43±0.12	5.32±0.2	9.23±0.34
T _max_ (h)	4.0	6.0	6.0
AUC0–t (μg ml ^−1^ h)	40.51 ± 1.16	96.34±3.7	116.61 ± 4.69
AUC0–∞ (ml ^−1^ h)	44.64 ± 2.76	91.43±5.3	158.34 ± 6.48
Elimination half-life (t1/2el) (h)	1.97 ± 0.10	2.34±0.5	3.49 ± 0.27
Elimination rate constant (Kel) (h ^−1^)	0.51 ± 0.004	0.2±0.006	0.18 ± 0.001
Clearance (Cl) (L h ^−1^)	0.55 ± 0.002	0.2±0.001	0.13 ± 0.002
Volume of distribution (Vd) (l)	1.62 ± 0.26	1.3±0.02	0.98 ± 0.01

Values are mean ± SEM (n = 3), C _max_-maximum plasma concentration, T _max_- time required to reach maximum concentration, AUC-area under curve, SEM- standard error mean.

### Pharmacokinetic parameters

**Figure 12.**
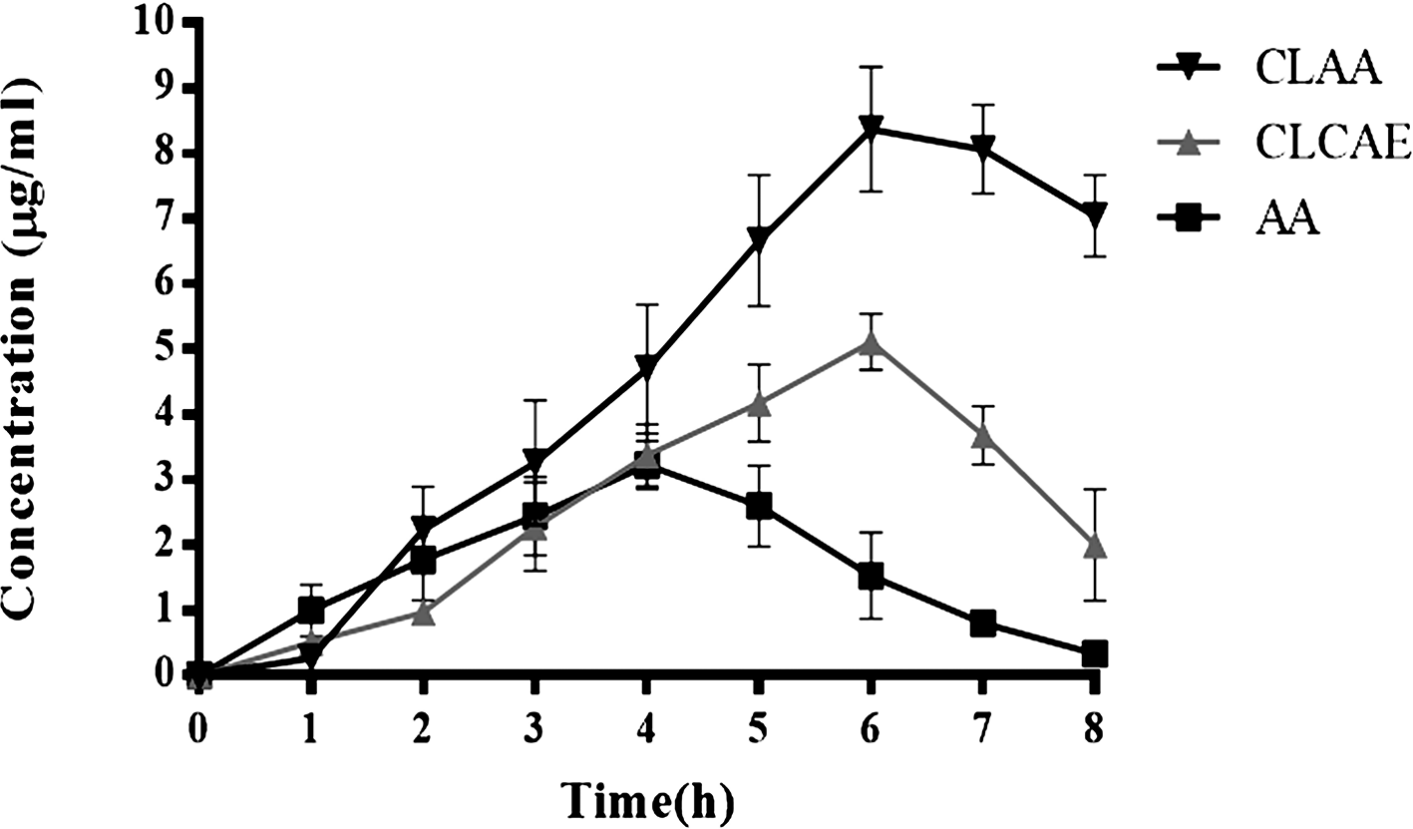
*In vivo* pharmacokinetic study of A) AA B) CLCAE and C) CLAA (n=6).

As shown in Table 5, the pharmacokinetic parameters were estimated using the computer software PK/PD ( Figure 12) (n =6). Tmax, Cmax, and elimination half-life values were greater in CLCAE and CLAA treated group than in serum from the AA-treated group. The optimised CLAA-treated rat serum showed lower values for the elimination rate constant, clearance, and volume of distribution. The optimised CLAA with a greater relative bioavailability of 75.56±0.6%, persisted for a longer duration in the body and the optimised CLCAE showed 53.23±0.3% relative bioavailability. A considerable improvement in the relative bioavailability of the CLAA took place as a result of the chitosan's bioadhesion activity, resulting in significant progress in the absorption of AA into the intestinal mucosa. In terms of time, there was a significant correlation between the rate of *in vitro* drug release and the *in vivo* plasma concentration of a drug. The formulations showed sustained drug release and a notable increase in drug bioavailability in the serum, possibly due to enhanced solubility and permeability compared to AA. The oral bioavailability of the optimised CLAA was significantly enhanced compared to optimised CLCAE and AA, which may be due to the decreased intestine and hepatic metabolism of the CLAA. ^85^^,^^86^ An experimental study showed that optimised CLAA carries enormous potential to enhance the efficacy of phytoconstituents with greater accuracy. However, more research into the clinical trial and *in vitro*- *in vivo* correlation (IVIVC) might help it get to market faster.

### Stability study

The formulation stability data at 5±2°C, 32±2°C/60% ± 2% RH is specified in Table 6. A stability study could not be carried out at higher temperatures (> room temperature) because phospholipids in liposomes would deteriorate at higher temperatures. ^87^ The results showed that after the third month, formulations kept at refrigeration temperature had better entrapment efficiency than all samples maintained at room temperature. The EE of optimised CLCAE varied with storage time and temperature. A considerable change in entrapment efficiency was observed and was found to be in the range of 51.3% to 49.5%, which indicated that liposomes were slightly stable. In contrast, throughout the stability study, the optimised CLAA exhibited a high degree of stability in entrapment efficiency. i.e., 71.2% to 71.2%. There was no difference in vesicle size in the optimised CLAA even after three months of storage (209.8 nm to 209.8 nm). The ζ potential of the optimised formulations exhibited minimal changes (+ 20.8 to + 22.2 mV), indicating excellent stability of liposomes. In the optimised CLCAE, a substantial difference was found in vesicle size compared to the CLAA. The vesicle size was slightly bigger after the second month of storage, which indicated that congeal liposomes subsequently affected the entrapment efficiency of the CLCAE.

**Table 6.** Stability study of the optimised CLCAE and CLAA by considering entrapment efficiency, vesicle size, and PDI.

	Entrapment study (%)							
	Initial		After 1 Month		After 2 Months		After 3 Months	
Optimised CLCAE And CLAA	5±2°C	32±2°C	5±2°C	32±2°C	5±2°C	32±2°C	5±2°C	32±2°C
	51.3±0.03	51.3±0.03	51.5±0.04	51.3±0.01	50.8±0.03	50.8±0.02	49.2±0.07	49.5±0.05
	71.2±0.03	71.2	71.2±0.03	71.0	71.2±0.03	71.2	71.2±0.03	71.2
	**Vesicle size (d. nm)**							
	224.4	224.4	224.7	224.8	224.2	224.4	234.2	234.7
	209.8	209.8	209.8	209.8	209.8	209.8	209.2	209.2
	**Zeta potential (mV)**							
	22.8	22.8	22.3	22.3	22.3	22.3	23.4	23.4
	20.8	20.8	20.8	20.8	20.8	20.8	20.8	20.8

**Special Characters (if applicable):**

AD = Alzheimer’s disease, CA = *Centella Asiatica*, AA = Asiatic acid, CLAA = Chitosan coated liposome of Asiatic acid, CLCAE = Chitosan coated liposome of *Centella Asiatica* extract, PM = Physical mixture, kDa = Kilo Dalton, NMR = Nuclear magnetic resonance, TEM = Transmission electron microscopy, AFM = Atomic force microscopy, PDI = Poly dispersity index, Tm = Melting point, C max = Concentration maximum, T max = Time maximum, RH = Relative humidity, °C = Degree Celsius, kHz = Kilohertz, Hz = Hertz, h = hour, cm = Centimetre, nm = nanometre AUC = Area under curve, μL = microlitre, mL = millilitre, N = normality, min = minute, mm = millimetre, μg = microgram, mV = millivolt, w/v = weight/volume, *p.o = * per oral, g = gram, *kg = * kilogram *,* AβPP = Amyloid beta protein precursor, CA1and CA2 = cornu Ammonis region 1 and 2, RH = relative humidity, BCS = Biopharmaceutics Classification System, DMF = Dimethylforamide, DMSO = Dimethyl sulfoxide, LD50 = Median lethal dose, Eq. = Equation, Fig. = Figure, n = number of trail, NTU = Nephelometric Turbidity Unit, DPPH = 2,2-diphenyl-1-picryl-hydrazyl-hydrate.

## Conclusion

The chitosan-coated liposomes of AA were found to be stable in the gastrointestinal tract GIT and controlled site-specific absorption. The developed novel product demonstrated an increase in AA's oral bioavailability compared to the conventional oral formulation as it showed great potential of extract when administered in modified form (CLCAE). CLAA also proved to be a better formulation than CLCAE because of AA’s uniform molecular size and potent pharmacological action. The CLCAE showed better pharmacological action than the standard extract of CA, attributed to the chitosan coating. Hence, the developed product can enhance efficacy and improve patient compliance through oral delivery. Furthermore, the modified, developed formulation can be commercialised as a nutraceutical product to prevent AD. The present work outcome is promising, and more experimental data and clinical study are required for further authentication of asiatic acid efficacy in nano-vesicular form for preventing or treating early stages of AD.

## Data Availability

Figshare: Data file 01 - In vitro evaluation data, https://doi.org/10.6084/m9.figshare.21484905.v1. ^88^ Figshare: Data file 02 - In vivo data, https://doi.org/10.6084/m9.figshare.21485256.v1. ^89^ Data are available under the terms of the Creative Commons Attribution 4.0 CC-BY (Attribution) Repository: The ARRIVE guidelines 2.0 checklist and flow chart for “Cationic biopolymer decorated Asiatic Acid and *Centella asiatica* extract incorporated liposomes for treating early stage Alzheimer’s disease: An *In-vitro* and *In-vivo* Evaluation”. https://doi.org/10.6084/m9.figshare.21621282. Data are available under the terms of the Creative Commons Attribution 4.0
