# Supplementary material for: Cationic biopolymer decorated Asiatic Acid and Centella asiatica extract incorporated liposomes for treating early-stage Alzheimer’s disease: An In-vitro and In-vivo investigation
Source: F1000Res. 2022 Dec 19;11:1535. [Version 1] doi: 10.12688/f1000research.128874.1 (PMC9887206; doi:10.12688/f1000research.128874.1)
Supplement: Peer review history

# Peer review: Reviewer response for version 1

**DOI:** 10.5256/f1000research.141508.r159533

**Published:** 2023-01-30

**Authors:** Udit J. Chaube

**Affiliations:** 1 Department of Pharmaceutical Chemistry, Institute of Pharmacy, Nirma University, Ahmedabad, Gujarat, India

This work aims to create and compare chitosan-coated liposomes of *Asiatic acid* (CLAA) and *Centella asiatica* extract (CLCAE) for increment in oral absorption and bioavailability in the treatment of ADs in early phases.

 The method of solvent evaporation was used by the authors for the development of CLAA and CLCAE.

 The ALCl _3_-induced Alzheimer's Disease Model was used to further assess this formulation, and the results showed that CLAA has better stability and was the optimal oral drug delivery mechanism.

 Although, there are certain gaps in the pharmacological evaluations which can be rectified. For Example: Missing of Scale on the Histopathological Slide.

 In the future, if the authors are carrying out this investigation, I urge them to employ Amyloid beta (A) rather than AlCl _3_ for the induction of AD. Additionally, the authors should use western blot analysis and gene expression studies in their upcoming research project (Explore Molecular Targets of Alzheimer's Disease), which would ultimately add innovation to the work. 

Is the work clearly and accurately presented and does it cite the current literature?

Yes

If applicable, is the statistical analysis and its interpretation appropriate?

Yes

Are all the source data underlying the results available to ensure full reproducibility?

Yes

Is the study design appropriate and is the work technically sound?

Yes

Are the conclusions drawn adequately supported by the results?

Yes

Are sufficient details of methods and analysis provided to allow replication by others?

Yes

Reviewer Expertise:

Cancer, Cancer biology, Design and Synthesis of Novel Leads as mTOR inhibitors and CDK2 inhibitors

I confirm that I have read this submission and believe that I have an appropriate level of expertise to confirm that it is of an acceptable scientific standard.

# Peer review: Reviewer response for version 1

**DOI:** 10.5256/f1000research.141508.r159536

**Published:** 2023-01-25

**Authors:** Manodeep Chakraborty

**Affiliations:** 1 Pharmacology Department, Himalayan Pharmacy Institute, Majitar, Sikkim, India

There is a need for more phytoconstituents-based studies in the pharmaceutical field.  The assessments of the research gaps are also self-evident, and this study's methodology and tools have a higher relevance to concluding the successful formation of the surface-modified liposome.

- Results of the pre-formulation study on AA comply with the standard reported literature limits. The developed RP HPLC method was found to be straightforward, quick, precise, reliable, and consistent for determining the AA (13 min). The performance qualities did not significantly alter during validation. 
- The conventional liposome of AA was developed using a solvent evaporation method. It was a well-accepted method for the preparation of liposomes. Chitosan was reported to be useful in coating negatively-charged liposomal surface because of electrostatic interactions between the negatively charged SPC and the positive charges of primary amino groups of chitosan.The ionotropic gelation method was adopted for coating the conventional liposomes.
- From the statistical optimization using Design Expert ^®^ software (3 ^2 ^full factorial designs) the % error between predicted and actual was found to be less than 5 %; the report was found to be reliable.
- In the H1 NMR investigation, the change in proton signals in the aromatic region clearly demonstrated the establishment of molecular bonds with AA. The phenolic region of AA and the combination of phospholipids have weak intermolecular contact. The average vesicle size of sonicated optimized CLCAE and CLAA were found to be 224.4 nm, 209.8 nm that signifies the optimum complex formation in which drug molecule tangibly bonded with the polar heads of lipid resulting in a reduction of surface negative charge and the vesicle size. The positive ζ charge of optimized CLAA shows good physical stability that ultimately favors the mucoadhesion property to the cell membrane. Studies using DSC and FTIR on CLAA that had been improved gave the first official evidence of the complexation between AA and SPC. TEM and AFM confirmed no evidence of aggregation or decomposition. But the scale was missing in the image. The optimized formulation had increased and sustained the drug release be due to physicochemical change and complexation between AA with SPC and chitosan.
- Alzheimer’s disease was successfully induced to experimental Wistar rats with the Alcl3 induced method which is a well-known method. The behavioral assessment was performed using the Y maze to confirm the disease progression. The scale in the histopathology image was missing. Both the histology examination and the neuronal count showed that the protection against disease progression was present.
- The results of the experiment showed that the group treated with the optimized formulation had a considerable amount of acetylcholine. This suggests that CLAA functions as an inhibitor of the cholinesterase enzyme.
- The results of the study are also openly admitted. The explanation related to the work was clearly written within its limitations and provides further direction and scope for research.

Is the work clearly and accurately presented and does it cite the current literature?

Yes

If applicable, is the statistical analysis and its interpretation appropriate?

Yes

Are all the source data underlying the results available to ensure full reproducibility?

Yes

Is the study design appropriate and is the work technically sound?

Yes

Are the conclusions drawn adequately supported by the results?

Yes

Are sufficient details of methods and analysis provided to allow replication by others?

Yes

Reviewer Expertise:

Molecular pharmacology

I confirm that I have read this submission and believe that I have an appropriate level of expertise to confirm that it is of an acceptable scientific standard.

# Peer review: Reviewer response for version 1

**DOI:** 10.5256/f1000research.141508.r159535

**Published:** 2023-01-11

**Authors:** Harishkumar Madhyastha

**Affiliations:** 1 Department of Cardiovascular Physiology, Faculty of Medicine, University of Miyazaki, Miyazaki, Japan

The manuscript entitled "Cationic biopolymer decorated Asiatic Acid and *Centella asiatica *extract incorporated liposomes for treating early-stage Alzheimer’s disease: An *In-vitro* and *In-vivo* investigation" by Dubey and others describes the conjugated polymers with nutraceuticals for combating early stages of AD. Investigation hypothesis is well-designed and all supporting evidences for preparations, characterizations and drug loading validation and finally functional evaluation of conjugates are being studied. Before final acceptance for indexing I recommend the authors to attend to the following points in the revision:

1. A hypothetical graphical abstract is required so that readers can quickly understand the whole theme in a single glance. 
2. Additional information on the rationale of using centella asiatica extracts is required in the introduction; especially on phyto-mapping constituents of this plan (which is required).
3. Did the investigators use whole plant including roots or some selective parts for extraction procedure?
4. The detailed procedures on formulation of conventional as well as chitosan-coated liposomes of CAE are required.
5. Replace figure 2 with peak only representations.
6. Scale bar is missing in TEM (Fig. 4) of CLCAE and CLAA.
7. Fig. 10: what is the magnification?
8. Some more points on the mechanism of actions of phytoconstitutents and AD is required in the discussion.

Is the work clearly and accurately presented and does it cite the current literature?

Yes

If applicable, is the statistical analysis and its interpretation appropriate?

No

Are all the source data underlying the results available to ensure full reproducibility?

Yes

Is the study design appropriate and is the work technically sound?

Yes

Are the conclusions drawn adequately supported by the results?

Partly

Are sufficient details of methods and analysis provided to allow replication by others?

Yes

Reviewer Expertise:

Nano medicine and Nutraceuticals

I confirm that I have read this submission and believe that I have an appropriate level of expertise to confirm that it is of an acceptable scientific standard, however I have significant reservations, as outlined above.

## References

[1] : GABA-enriched teas as neuro-nutraceuticals. *Neurochem Int* .2020;141: 10.1016/j.neuint.2020.104895 104895 10.1016/j.neuint.2020.104895 33144101
